# Status quo and directions in deep head and neck hyperthermia

**DOI:** 10.1186/s13014-016-0588-8

**Published:** 2016-02-11

**Authors:** Margarethus M. Paulides, Gerda M. Verduijn, Netteke Van Holthe

**Affiliations:** Erasmus MC Cancer Institute, Box 5201, 3008AE Rotterdam, The Netherlands

**Keywords:** Head and neck cancer, Radiofrequency, Hyperthermia, Electromagnetic modelling, Thermal therapy

## Abstract

The benefit of hyperthermia as a potent modifier of radiotherapy has been well established and more recently also the combination with chemotherapy was shown beneficial. Also for head and neck cancer, the impact of hyperthermia has been clinically demonstrated by a number of clinical trials. Unfortunately, the technology applied in these studies provided only limited thermal dose control, and the devices used only allowed treatment of target regions close to the skin. Over the last decade, we developed the technology for deep and controlled hyperthermia that allows treatment of the entire head and neck region. Our strategy involves focused microwave heating combined with 3D patient-specific electromagnetic and thermal simulations for conformal, reproducible and adaptive hyperthermia application. Validation of our strategy has been performed by 3D thermal dose assessment based on invasively placed temperature sensors combined with the 3D patient specific simulations. In this paper, we review the phase III clinical evidence for hyperthermia in head and neck tumors, as well as the heating and dosimetry technology applied in these studies. Next, we describe the development, clinical implementation and validation of 3D guided deep hyperthermia with the HYPERcollar, and its second generation, i.e. the HYPERcollar3D. Lastly, we discuss early clinical results and provide an outlook for this technology.

## Introduction

In current clinical practice, tumors of the head and neck (H&N) region are treated with surgery, radiotherapy (RT), chemotherapy or combinations of these. Early cases are usually treated with either surgery or RT, where locally-advanced primary carcinoma of the H&N are treated with RT, often combined with chemotherapy [1]. Following the introduction of hyper-fractionation schedules of RT and technological improvements like intensity modulated RT (IMRT), higher tumor dose and reduction of some toxicity endpoints, such as xerostomia [2], can be achieved but the toxicity remains substantial [3, 4]. In 2000, Pignon et al. [5] published a three meta-analyses study, which showed that combining RT with simultaneous chemotherapy on average results in an 8 % absolute increase of the overall 2-years survival. More recently they observed a 4.5 % absolute benefit at 5-years in another meta-analysis involving 87 trials [6]. Still, improvement is warranted since loco-regional recurrence rates are up to 30–60 % after complex multimodality treatment [7]. In addition, more than 80 % of patients experience severe toxicity with current regimens, as well as enduring long-term effects from treatment, relapse, or metastasis [1]. For chemotherapy, the reported improved treatment outcomes were accompanied by enhanced systemic toxicity or exacerbate local tissue reactions, while accelerated fractionation causes high rates of severe mucositis resulting in early stoppage of randomized trails [8]. Hence, this combination often is too toxic, in which RT alone provides a 2-years loco-regional control of only 50 % and an overall survival of 53 % [6]. In recurrent H&N cancer, 80 % of patients are not eligible for a curative treatment and local tumor control after retreatment is only 26–52 % [9, 10] leading to a 2-years overall survival of 10–20 % for reirradiation and chemotherapy [11]. Moreover, toxicity after re-treatment with concurrent irradiation and chemotherapy is even more severe (grade four toxicity 18–23 %, and grade five toxicity 5–8 %) [11, 12]. Hence, as most patients die because of local treatment failures, there is a need for improved loco-regional treatment.

Biological research showed that hyperthermia (HT), elevation of the tumor temperature to 40–44 °C for 60–90 min, is amongst the most potent modifiers of RT known today [13]. In addition, HT also enhances the effect of many chemotherapies [14]. The impact of HT without inducing extra toxicity was clinically demonstrated by a number of clinical trials, including four studies in H&N cancer. Unfortunately the technology used in these studies allows heating of only superficial regions. In addition, the technologies and strategies applied provide limited control of the applied thermal dose. This makes it difficult to reproduce the results or to quantify the level of temperature rise or thermal dose required [15]. Hence, although these trials demonstrated statistically significant outcomes, the clinical objective in terms of required temperature or thermal dose cannot be quantified. This forms a hurdle to reproduce the applied dose, which hampers progress through follow-up clinical trials and dissemination of the treatment to other clinics. Therefore, over the last decade, we developed technology to apply deep HT to all regions in the H&N region, while in the meantime also providing the most objective and reproducible treatment strategy achievable. In this way, the means are provided to mature and popularize the application of deep HT in H&N cancer.

The current publication provides an overview over the results obtained in phase III clinical studies and reviews the equipment and dosimetry applied. Next, we review the development, clinical implementation and validation of simulation guided application of deep HT to target regions in the H&N region using the Rotterdam approach. Hereto, we summarize the technology developed (the two applicator versions and treatment planning), the clinical experience and heating quality obtained and the early clinical outcomes.

## Review

### Status quo in head and neck hyperthermia

There are four prospective randomized phase III trials that demonstrate the effectiveness of HT in H&N tumors. Table 1 summarizes the results per study, where more extensive reviews concerning the clinical results are provided in [16] [17]. In addition, we summarize the most important study, heating and dosimetry details for these studies. Lastly, we included the results of the most recent study by Datta et al., whom overviewed the results of not only these four randomized phase III trials, but also included well-controlled non-randomized trials on HT for several tumor sites, including the H&N.

**Table 1:** Results of randomized phase III trials on hyperthermia for cancers in the head and neck

Reference	Tumor	Combi	N	Endpoint(s)	-HT	+HT	Heating	Quality control
Valdagni et al. 1988 & 1994 [18, 19]	Neck Nodes	RT	44	CR	41 %	**83** %	Radiative (280–300 MHz)	Invasive, > 4 each HT session (periphery:core = 4:1)
				5 years LC	24 %	**69** %		
				5 years OS	0 %	**50** %		
Datta et al. 1990 [20]	OC, OP (stage I–IV)	RT	65	CR	31 %	55 %	Capacitive, (27.12 kHz)	-
Huilgol et al. 2010 [21]	OC, OP, HP (stage II–IV)	RT	54	CR	42 %	**79** %	Capacitive (8 MHz)	Invasive (infrequent)
Hua et al. 2011 [22]	NP (stage I–IV)	CRT	180	5 years LC	79 %	**91** %	Conduction (resistive wire)	Nasal cavity internal skin temperature
				5 years PFS	63 %	**73** %		
				5 years OS	70 %	78 %		
Zhao et al. 2014 [23]	NP (stage II–IV)	CRT	83	3-years OS (QoL)	54 %	**73** %	Capacitive	Nasal cavity internal skin temperature

*RT* Radiotherapy, *CRT* Chemo-radiotherapy, *N* total number of included patients in the study, −*HT* results without HT, *+HT* results with HT, *LC* local control, *CR* complete response, *PFS* progression free survival, *OC* oral cavity, *OP* oropharynx, *HP* hypopharynx, *NP* nasopharynx. Results in bold are significant at the 5 %-level. Toxicity was comparable in all randomized trials, although Zhao et al. found an improved quality of life (QoL)

Valdagni et al. [18, 19]

*Study design:* Fixed, inoperable and previously untreated metastatic lymph nodes, from a H&N or unknown primary, were randomized to receive RT or RT combined with HT. In total, 44 N_3_ metastatic lymphnodes in 41 patients were included and the randomization was performed per individual node. Fractions of 2.0–2.5 Gy were applied five times per week to a total dose between 64–70 Gy, average dose was 68 Gy for RT alone and 67.5 Gy for RT + HT. In the combined treatment arm, nodes were further randomized to receive two or six heat sessions: either two sessions of HT in the first week of radiation or six sessions in the first three weeks of radiation. Heat sessions were applied 20–25 min after RT, with an interval of at least 72 h between subsequent heat sessions.

*Outcomes:* In an interim analysis, prematurely a significant improvement in complete response rate after RT + HT was seen versus RT alone, 82.3 % vs 36.8 % (*p* = 0.0164). Note that, since the study was prematurely closed for ethical considerations, the number of patients accrued was limited. Follow-up analysis showed that the statistically significant difference between “early” response rate in the two arms translated into a similarly significant long-term difference in actuary nodal control (*p* = 0.015) and 5-year survival rate (*p* = 0.02) [19]. They also randomized between two and six heating sessions in the combined HT and radiation group. A small trend towards six sessions, but no significant advantage could be demonstrated. In this study, the addition of heat did not result in any enhancement of early or late side effects on normal skin, the only exception being one blister as a consequence of superficial overheating because of metastatic skin involvement.

Second study: In addition to previous study using the same scheduling, Amichetti et al. [20] showed in a I/II trial that HT also improves treatment outcome when added to a hyper-fractionated radiation schedule.

*Heating and QA:* Superficially located metastatic lymph nodes were heated using radiative microwave heating using the MA 150 applicator (280–300 MHz, aperture size 10 x 13 cm) of the BSD 1000 system (BSD Medical, Salt Lake City, USA). For each treatment, invasive thermometry was performed using multipoint Bowman thermal probes in a minimum of five intra and peritumoral locations and on a minimum of three sites on the skin. Twelve thermal probes were selectively inserted into tumor-normal tissue interfaces, with a nodal periphery-nodal core ratio of approximately 4:1. Thermal mapping along the probe axis was occasionally performed. Probe localization and verification was mostly determined from orthogonal x-ray films. The goal for each session was to attain a tumour temperature of at least 42.5 °C per 30 min at the periphery of the target volume.

Datta et al. [21]

*Study design:* Sixty-five patients were included in a randomized clinical study to evaluate the efficacy of local HT as a concomitant agent to RT. Primarily advanced tumors were included, but the majority of those were close to the skin and 14/33 tumors in the cheek. All the patients were treated with 50 Gy telecobalt therapy in 5 weeks by using two parallel, opposed fields encompassing the primary and the regional lymphatics. A boost of 10–15 Gy in 5–10 days was given to the gross tumor volume. Ten HT sessions were applied to the gross tumor volume twice a week with a period of 72 h between the two sessions. RT was applied immediately after heating.

*Outcomes:* Overall no significant increase in response rate was observed. In patients with Stage I and II, response was similar, while patients with advanced disease (Stage III and IV) had a significantly better tumour control after the combined treatment. Complications, especially skin and mucosal reactions, of both groups were almost identical in type and magnitude.

*Heating and QA:* Capacitive heating at 27.12 kHz was applied by the Siemens Ultraterm 607E diathermia machine. Two rubber pad electrodes were placed on either side of the tumour with a felt pad placed between skin and electrode to act as a coupling medium. Thermometry was measured using a thermocouple placed in the center of the tumour and measuring at 15 min intervals while switching off the heating device. Power was increased until a temperature of 42.5 ± 0.5 °C was achieved, which was maintained at least 20 min.

Huilgol et al. [22]

*Study design:* A total of 56 patients with cancers of the oropharynx, hypopharynx and oral cavity were randomized to receive either RT or RT–HT. Primarily T3-T4 stages were included and all patients were treated with radiation to a dose of 66–70 Gy in 6.5–7 weeks. Patients in the study group were treated with weekly 30 min HT applied after RT at an unknown interval. HT was stopped if patients developed grade II or higher thermal burns.

*Outcomes:* Complete response was seen in 42.4 % of RT alone group compared to 78.6 % in the HT group (*p* < 0.05). Twenty-three patients could finish more than five sessions, where five dropped early due to pain or systemic stress. Overall, acute and late toxicities were comparable in both treatment arms except for an overall increase of thermal burns in the HT group.

*Heating and QA:* HT was delivered on modified Thermotron RF-8 (Vinita Yamamoto Inc., Japan), operated at frequency of 8 MHz. Ten minutes precooling was applied before starting HT. Guided by visible tumor or anatomical landmarks, both antennas were placed on each side of the neck. After impedance matching, power was gradually escalated till the patients complained of unbearable pain, stress or discomfort. Power was then reduced and maintained till completion of the treatment. Input power varied from 400 to 1000 kW. Patient specific quality assurance was not performed, but in some patients an average temperature in the tumor of 42.3 °C was reported as measured using invasive thermometry with a thermistor probe. In addition, the performance of the applicator was investigated [23]. These experiments showed that this device provides an option for lateral tumors but not deeply located, i.e. despite the facts that the phantom cylinder was much smaller than that in other studies (10 cm vs 12–15 cm [24]), that fat was not modelled and vicious cooling was applied, still substantial heat was absorbed only in superficial tissues.

Hua et al. [25]

*Study design:* In total 180 patients presenting with nasopharyngeal cancer were randomized to receive (chemo)-RT with or without HT. A total dose of 70 Gy was administered to the primary tumour and the upper neck in 2Gy fractions, with or without a boost applied using brachytherapy. Lymphnode levels were always irradiated by at least 50 Gy and clinically positive neck nodes received a minimum dose of 60 Gy. RT was delivered daily, five times a week. All T3 and T4 tumors were treated with concurrent chemotherapy consisting of cisplatin and 5-FU. HT was applied to the tumor once a week within 30 min before or after RT, for a total of seven times, or 2 h after cisplatin finished.

*Outcomes:* This study demonstrated a significant improved CR, i.e. 81.1 % (CRT) to 95.6 % (CRT + HT) (*p* = 0.003). After 5-years local control (p = 0.022) and PFS (*p* = 0.039) were significant but OS was not significant (*p* = 0.14). Subgroup analysis indicated that RT was already sufficient in for T1 tumors, but that HT had a significant contribution to local control of T2/T3 cases, although no significant overall survival advantage could be demonstrated. Possibly because tumors with skull base invasions were excluded and their consequent low number, no significant improvement for T4 tumors could be established. However, the average improvement by HT was also smaller, which suggests problems in achieving sufficient temperatures in these larger tumors. The most common toxicities were local mucositis, erythema, and blisters, but acute oral mucous toxicity in both arms was comparable and no lethal toxicity occurred. Late adverse reaction was comparable in the HT group.

*Heating and QA:* Specifically designed intracavitary equipment was used based on a commercially available device (WE2102-A Microwave HT System, Yuan De Biomedical Engineering, Beijing). The technique employed was mentioned to be 915 MHz microwave radiation [25], but the reply to a letter to the editor [15] mentions a hot wire based conductive heating technique [26]. Patient specific quality assurance was performed in this study by measuring the temperature of the nasal cavity mucosa using a thermocouple temperature sensor. In addition, an experiment with a beagle dog was performed to relate skin temperatures around 43 °C to temperatures 3 cm into the mucosa 42.7 °C. Unfortunately, it remains unclear if the dog was anaesthetized, possibly leading to a downregulated thermoregulatory response and higher temperatures achieved.

Zhao et al. [27]

*Study design:* In total 83 patients presenting with nasopharyngeal cancer were randomized to receive (chemo)-RT with or without HT. First,

chemotherapy (paclitaxel 135–175 mg/m^2^, cisplatin 60–90 mg/m^2^, every 3 weeks, for two cycles) was given. Second, the physical condition of the patients was evaluated. One week later, patients received 2 Gy fractions conformal radiation therapy (3D-CRT) to the cervical lymphnodes (5 weeks, 50Gy) and to the nasopharynx target (7 weeks, 70–74 Gy). All patients received concomitant chemotherapy (cisplatin 30 mg/m^2^, once a week). Third, patients received chemotherapy (paclitaxel 135–175 mg/m^2^, cisplatin 60–90 mg/m^2^, every 3 weeks for four cycles). Patients randomly selected for the study arm received capacitive HT for one hour every other day after RT, for a total of 21 times in 7 weeks.

*Outcomes:* The 36-month survival rate was 73 % for CRT + HT compared to 54 % for CRT (*p* = 0.041). The average disease-free survival time was 48 months versus 37.5 months in the reference arm (*p* = 0.048). In the post-treatment questionnaires, several NPC-specific treatment side effects (pain, swallowing, speech, social eating, opening mouth, dry mouth, sticky saliva) were asked. These quality of life (QoL) domains were better preserved with CRT + HT compared to CRT at different time-points.

*Heating and QA:* In this study, the commercially available HG-2000-type non-invasive

capacitive radiofrequency applicator (Zhuhai Hokai Medical Instruments, Guangzhou, China) was used. The frequency of this devise is reported to be 13.56 MHz or 40.68 MHz. The diameter of the primary plate was 0.8 cm, the length 1.5 m; and the maximum output power was 1200 W. The temperature in the tumor was estimated by measurements at the internal skin surface. Power was increased from 100–150 W every 10 min by 10–20 W steps until the surface temperature reached 43 °C.

Overview of four randomized and four non-randomized studies: Datta et al. [28]

*Study design:* An overview of the status quo on HT in terms of response rate for (chemo)-radiation with or without HT [28]. For the H&N, this study included in total 717 patient from four of the above mentioned randomized phase III studies, and four non-randomized studies for which representative historical control groups were available.

*Outcomes:* This review demonstrated a statistical significant difference (*p* < 0.001) in favor of the HT group, i.e. complete local response was 50.3 % (183/364) for (chemo)-radiation versus 75.3 % (266/353) for (chemo)-radiation combined with HT, with an odds ratio of 3.71 (95 % CI, 2.55–5.38).

*Heating and QA:* In 2/8 studies, intracavitary HT was used by exploiting either resitive wire or microwave HT. 3/8 studies used capacitive HT at frequencies between 8 and 13.56 MHz. In 3/8 studies, radiative HT was applied at frequencies between 280 and 915 MHz, although of one study the frequency could not be traced. Besides the quality assurance described above, no invasive thermometry was used to control the heating.

In summary, there is accumulating evidence that HT improves radiation and chemotherapy in H&N cancers. In addition, no extra toxicity has been observed. One study even showed an improvement in RT affected quality of life (QoL). Still improvements are warranted because the commercially available applicators only provide heating of lateral tumors while adequate heating of the complete CTV, i.e. tumor and lymphnode stations affected and/or at risk, usually requires heating large regions that can also extend to regions deeper than 4 cm from the skin. Note that, although current trials have shown the greatest benefit in large-hypoxic-tumors, HT may also have great future potential specifically in this CTV-GTV margin due to its selective effect on tumor cells stemming from the sequential application of RT and HT [29].

Since the application of HT in the phase III trials is characterized by very limited monitoring and control of the applied thermal dose, it is difficult to reproduce the results, or to quantify the level of temperature rise or thermal dose required. Hence, although these trials demonstrated statistically significant outcomes, the clinical objective in terms of required temperature or thermal dose has not been established. Consequently, progress through follow-up clinical trials has been poor and dissemination of the treatment to a larger group of clinics was hampered.

To overcome the hurdles of deep heating and control, over the last decade, we developed technology to apply deep HT to the CTV combined with the most objective and reproducible treatment strategy achievable based on pre-treatment planning simulations.

### Equipment for controlled deep hyperthermia in the head and neck

#### The HYPERcollar: in use from 2007–2014

**Fig. 1:**
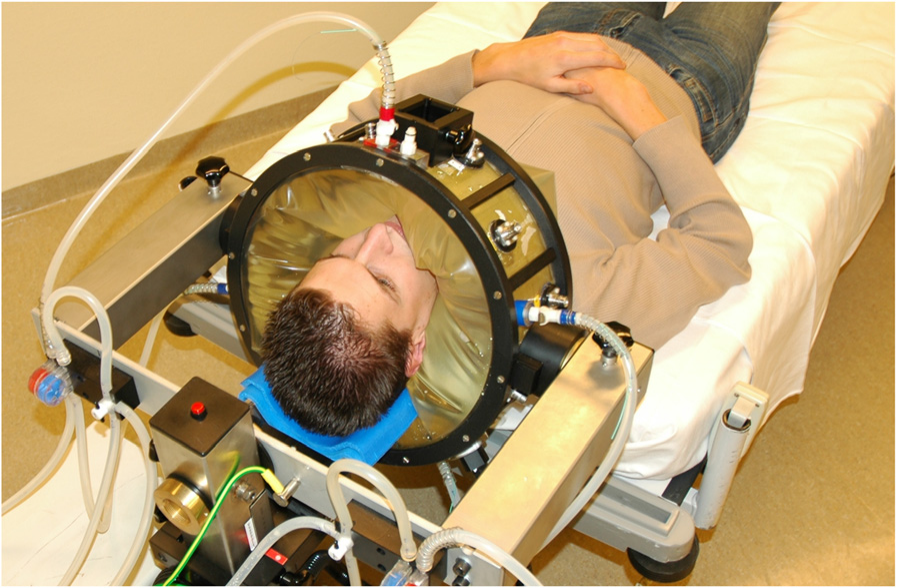
The HYPERcollar applicator, as it was used until 2014, surrounding the 1^st^ author. This applicator features twelve antennas fed by a high power system with twelve channels with independent power and phase control for focusing the heat at the target region

The design of the HYPERcollar (Fig. 1) differs from historic design approaches in the fact that we worked inverse. That is, we first defined the target volume to be heated and then relied completely on theoretical modeling to design the applicator to meet the defined objectives in terms of power absorption, expressed in the specific absorption rate (SAR, W/kg). An important advantage of this approach is that a priori the translation of predicted SAR distributions for such theoretically designed applicators have a much higher probability to resemble the actual SAR distribution in vivo, than existing devices where the electromagnetic model of the applicator was drafted afterwards. For the design of the HYPERcollar, extensive parameter studies were performed starting with a simple arrangement and stepwise increasing the complexity of the model. We showed that frequencies in the range of 400 to 600 MHz can focus energy efficiently into the center of the neck and selected 434 MHz as best compromise [24, 30]. At this frequency, increasing the number of antennas and antenna rings led to a better focusing of the power [31, 32]. To enable heating patients of variable size with a centrally located tumor, i.e. the worst-case scenario, we selected an arrangement consisting of two rings of six antennas with a radius of 20 and 6 cm spacing between the two ring arrays of applicators. A crucial step in the HYPERcollar applicator design was to replace the dipole antennas for a resonant patch antenna [33]. The direct feeding of the patch antenna by a coaxial cable without requiring a matching circuit leads to greater robustness and higher efficiency, but also easier and more accurate modeling. Consequently, the predicted results were in very good agreement with the experimental results, for the electrical performance as well as the SAR distributions [31]. SAR validation was performed with a specially constructed laboratory prototype H&N applicator, including a neck mimicking cylindrical muscle phantom [24]. Using phase steering, we showed that the antenna arrangement enables to adjust the SAR focus in radial (x/y) and axial (z) directions. A central 50 % iso-SAR focus of 35 mm (±3 mm) in diameter and approximately 100 mm (±15 mm) in length were obtained for all investigated settings. For the HYPERcollar, we found identical values [31] and demonstrated the ability of the HYPERcollar applicator to adequately deposit microwave energy in the target region in a simulation study using a representative clinical case [34].

#### The HYPERcollar3D: in use since 2014

**Fig. 2:**
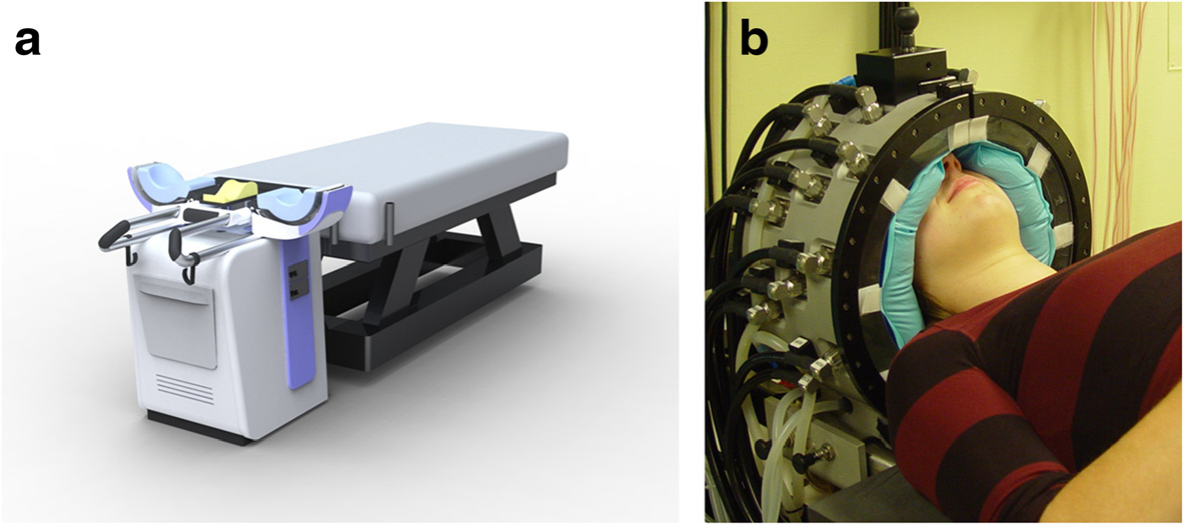
**a** Design and (**b**) clinical prototype of the HYPERcollar3D applicator. Twenty antennas are arranged in three rings to allow more precise longitudinal steering, as compared to the HYPERcollar applicator. In addition, positioning reproducibility and waterbolus shape stability was improved to improve the focusing ability and reproducibility of the applicator, and hence the treatment. Note that still a high power system with twelve channels is used and hence a dedicated antenna selection procedure was developed

The clinical experience obtained with the HYPERcollar revealed crucial information on how to proceed in targeted heating of H&N tumors [35]. Unbiased observations of the use of equipment and protocols and interviews with different stakeholders, i.e. patients, technicians, physicists and medical doctors, were used to reveal demands or latent needs. We found that a major item in fast, accurate and comfortable application of H&N HT, especially simulation guided, is the controllable and reproducible positioning of the patient which proved difficult using the HYPERcollar [35]. Additionally, the shape of the water bolus was difficult to reproduce while we found that it has strong effects on the treatment performance with respect to the maximum applicable RF-power, the reflected power for some specific antenna’s, and the occurrence of superficial hot spots. Finally, the applied amplitude and phase settings revealed that the SAR distributions could be improved by optimizing antenna positions. Therefore, we fully redesigned the HYPERcollar, leading to the HYPERcollar3D [36], of which the most improvements are:

*Patient positioning:* In the new design (Fig. 2), patient positioning has been improved by implementation of a standardized headrest, as used in RT, combined with a laser alignment system to enable accurate reproduction of the position during the planning CT. Tests with five volunteers demonstrated that this procedure facilitates a positioning accuracy (full-range) of ±2 mm (ventral-dorsal), ±4 mm (cranial-caudal) and, although not validated, an estimated ±4 mm (left-right) [37].

*Waterbolus:* The HYPERcollar3D design utilizes a smaller antenna-skin distance, splitting functionalities in an inner (flexible) and outer (stiff) waterbolus to substantially improve waterbolus shape robustness. To account for the differences in anatomy, the flexible part of the bolus can be replaced by other inserts to enable treatment of tumors in the neck (convex) as well as in the head (concave). The improvements were validated using a specifically designed mock-up applicator and CT scanning was used to verify that the waterbolus closely conformed to the skin contour. In addition, a high resolution infra-red camera was used to show that the temperature in the waterbolus was homogeneous, which demonstrates the homogeneity of skin cooling. Hence, this modular and predictable waterbolus shape substantially improves the accuracy of translating HTP from screen to patient.

*Antenna array arrangement:* Simulation studies in SEMCAD-X (version 14.8.5, Schmid & Partner Engineering, Zurich, Switzerland), and 3D patient models of 26 patients treated with H&N HT, were used to analyze the predicted HT treatment quality (hotspot target quotient: HTQ [38]) for various antenna array arrangements [39]. We showed that a higher number of antennas (20 vs 12), and their repositioning, provides a substantial reduction of hot-spot importance, i.e. HTQ reduces by 32 %. In addition, an average of 981 W can be used, which drastically reduces the probability for system power to become a treatment limiting source. Combined, these improvements result in a predicted two-fold increase in SAR level that can be clinically applied to the target [39].

In summary, the HYPERcollar3D provides accurate patient positioning and improves water bolus (WB) shape stability and tissue connection. Combined, the improvements in positioning and the more reproducible waterbolus shape improve the translation of simulation settings into the clinic, which improves the SAR focusing and makes the simulation guided HT more effective. Besides improved accuracy, the renewed positioning strategy also allows for quicker patient positioning and increased patient comfort. Note that a comfortable position is not only desired from a comfort point of view but also aids in a reproducible positioning.

#### Applicator modelling and quality assurance

Simulation accuracy is always critically dependent on a sound implementation of the applicator into the electromagnetic simulator. Hence, extensive validation of the model is required to match the performance of the applicator in the simulator and in reality.

These 3D CAD implementations are also used in the HTP process to predict the electric field, and hence the SAR distribution when the fields of antennas are combined, for each patient [36]. During the development of the HYPERcollar and HYPERcollar3D, we already developed and validated the EM implementation of these applicators. In addition, stable performance of the applicator over time must be verified to ensure the predictive value of the simulator, and hence the effectiveness of a simulation guided treatment strategy. Simultaneous to the development of the HYPERcollar3D, we therefore also extended our quality assurance protocol. Initially, quality measurements were performed by measuring the electric field sensors and infra-red thermometry (temperature increase profile) using a split-phantom [31]. Hereto, we developed a dedicated setup for 3D scanning of the electric field distribution produced by the applicator [40]. In this way, the 3D electric field distribution can be measured in tissue simulation liquids inserted into a cylindrical “neck-sized” casing with a diameter of 15 cm. This procedure is still under development, especially obtaining a realistic waterbolus shape is cumbersome, but already a match between measurement and simulations better than 10 % in electric field was obtained. By comparison of the normalized root-mean-squared measured and simulated electric field distributions, we showed that the measurements qualitatively matched very well to the simulations for the HYPERcollar3D, i.e. the maximum SAR could be predicted < 5 mm accuracy. Still, initial measurements did not fulfill our gamma criterion (dose difference < 10 %, distance to agreement < 10 mm). Note that disagreements were at locations outside of the volume of interest, i.e. in the region beneath the waterbolus edge. However, by using the 3D reconstruction of the waterbolus shape, a better match between simulations and measurements was obtained leading to approval for clinical use [41].

#### High-power amplifier and control system

In so called “phased array applicators”, like the HYPERcollar and HYPERcollar3D, the most important treatment parameters to be optimized are the properties of the microwave signals applied to the antennas, i.e. the power and phase of the signals supplied by the high power amplifier and control system. Hence, very important in our simulation guided approach is an accurate transfer of optimized signals into the clinical situation. Therefore, we developed and rigorously tested a 433.92 MHz multi-channel amplifier system with accurate control over the amplitudes and phases applied to the antennas [42]. The design consists of a direct digital synthesizer (DDS) system that generates 12 phase-controlled coherent 433.92 MHz signals, which are amplified to maximum 200 W output per channel. Directional couplers are placed at the amplifiers to couple a small portion of both forward and reflected signals to gain-and-phase detectors. The power setting is applied with a resolution of 2 W and for the phase it is 0.1°. In addition, the sampling rate of 100Hz also allows fast switching approaches to homogenize the heating pattern. The performance of the designed amplifier system was tested by measuring the RF spectrum, power and phase accuracy, and by characterizing the feedback control. The measurement accuracy for the power (< 5 %) is valid for at least 20 days after calibration and for the phase (< 5 °) it is valid for at least 2 months. Hence, this system provides the required technology for accurate conversion of optimized settings into the clinic.

### Pre-treatment planning

**Fig. 3:**
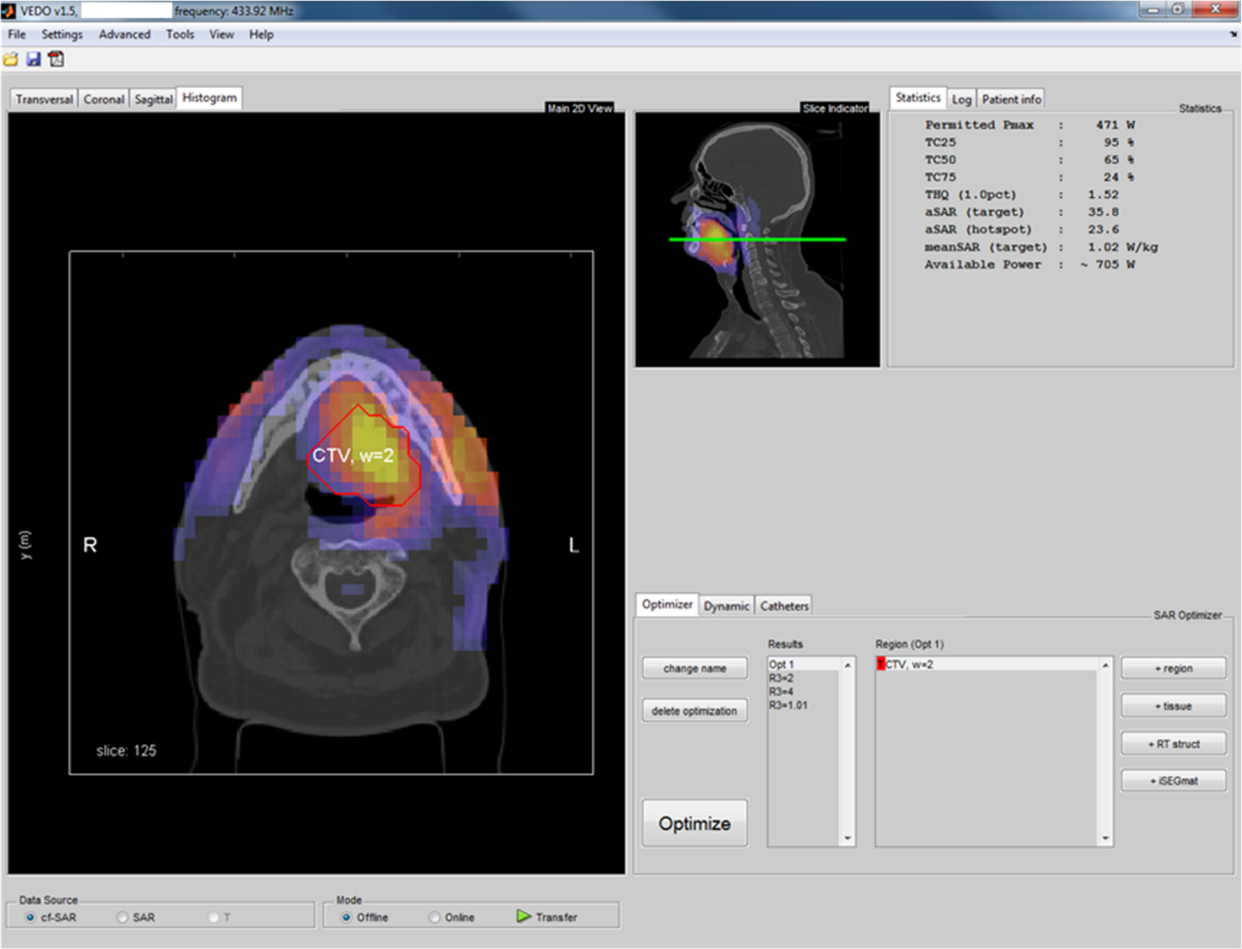
VEDO interface displaying the predicted SAR (or temperature) distribution on top of the planning CT in all Cartesian directions. VEDO also provides an implementation of the complaint adaptive steering approach. In addition, VEDO provides indicators to assess treatment quality based on the simulated SAR as well as to assess the risk of overtreatment

HT treatment planning (HTP) is the process of deriving a 3D patient model and using 3D electromagnetic and temperature simulation tools to optimize treatment parameters such that the best achievable temperature pattern in the clinic is obtained [43]. Simulation studies have shown that HTP is an absolute requirement for clinical use of the HYPERcollar [34]. The latter is even more emphasized as in general, interstitial thermometry in H&N tumors is in 70 % of cases deemed not feasible [35]. The required catheters that are inserted into tissue are a burden to the patient and their discomfort leads to their removal usually after one or two sessions [44]. In addition, the temperature measurement probes inserted into these catheters, even when applying multi-sensor probes, provide only limited sampling of the temperature distribution. Hence, it is clear that optimizing HT treatment quality on temperature measurements alone has a poor potential. This is why, from the start of H&N HT we have focused on using HTP not only for treatment optimization, but also for dosimetry purposes. Hereto we developed VEDO (Visualizer for Electromagnetic Dosimetry and Optimization, Fig. 3) [36]. Based on the electric field and tissue distribution generated using dedicated HTP simulation software, like Hyperplan or SEMCAD-X, VEDO can optimize treatment settings to maximize the cubic filtered SAR (cf-SAR: [36]) in pre-treatment planning. In addition, VEDO provides real-time re-optimization possibilities for adaptive application of HT.

#### Patient modelling

A major element in an accurate simulation of the SAR distribution is the accuracy of the patient model. Although new strategies are underway that allow creation of inhomogeneous patient models [45], tissue volumes are segmented using a computed tomography (CT) scan in our current protocol. Literature based homogeneous properties are assigned to these tissue volumes to create a 3D dielectric property distribution. Initially, the segmentation process for normal tissues was semi-automatic, i.e. areas of exterior, fat, muscle and bone were segmented automatically and tissues such as cartilage, white matter and grey matter were manually discriminated. This process took 6–8 hours for a complete 3D model. Therefore, we developed a software tool that uses the information of earlier segmentations for discriminating tissue types for the CT of the patient to be treated [46]. We showed that segmentation accuracy of this automatic procedure falls within the variation observed when a CT scan is segmented by different observers. By a simulation study, we showed that the uncertainty of SAR predictions caused by segmentation errors can be neglected in the total modelling uncertainty [47]. In addition, we studied if simulation accuracy can be improved by incorporating also the information from magnetic resonance imaging (MRI). Hereto, we developed a dedicated matching tool to align the CT and MRI scans [48], and extended and validated the auto-segmentation tool [49]. Next, using a simulation study, we showed that additional segmentation detail is not important for the signal optimization [50]. However, when predicting the SAR in organs at risk, the number of segmented tissues was shown relevant, i.e. a difference in maximum SAR of 10.9 % was predicted for CT-based (homogeneous) brains versus MRI-based (white matter, grey matter, cerebral spinal fluid) modelling [51]. Moreover, in specific cases, segmentation detail was required to accurately predict hotspots [50].

In addition to segmentation of normal tissues, also the tumor tissue must be delineated since it has different EM and thermal properties. In current clinical practice, this is done manually. From the beginning, we have focused on resembling the RT treatment, and hence the imaging and patient positioning during the HT treatment. This allows to directly take over the delineation of the target volume. Initially, only the clinical target volume (CTV) was performed by the radiation oncologist, and hence tumor properties were assigned to the entire target region. Later the protocol was improved to also include the segmentation of the gross tumor volume (GTV), representing the tumor mass visible on the CT and MRI scans. Since conductivity values in the tumor are assumed to be higher [34], this led to more accurate, but lower SAR predictions in the target area. Note that the values of the dielectric properties in tumor, and their heterogeneity, are still unknown and subject of investigation.

#### Optimization and treatment quality quantifiers

In HT treatment, maximization of the target temperature, as quantified by a range of descriptors like the median temperature over time and space, i.e. T50 [52], is aimed for. However, the accuracy of temperature simulations required for optimization of these quantifiers is hampered by the strong uncertainties and variations in thermal tissue properties [45]. Hence, in the current clinical routine we use the target-hotspot quotient (THQ), or alternatively the hotspot-target quotient (HTQ = 1/THQ) [36], since this parameter correlated with simulated temperatures for deep pelvic HT. THQ quantifies the mean SAR supplied to the target versus the mean SAR in the highest percentile of normal tissue, i.e. the SAR hotspots. This parameter is used in the optimization procedure to maximize treatment quality. In addition, we use the SAR coverage of the target at 25 % of the maximum value in tissue, i.e. TC_25%_ [36]. TC_25%_ is used to decide whether a H&N HT treatment is possible:

-For TC_25%_ ≥ 75 %, always a HT treatment was prescribed.
-For TC_25%_ in the range of 25–75 %, thermal catheters were a requirement to verify treatment quality.
-TC_25%_ below 25 % was interpreted as insufficient heating and HT was not applied in these patients.

Here, we anticipated that 75 % coverage at the 25 % iso-SAR level would be adequate to induce a sufficient temperature rise in the entire target when taking into account thermal conduction. Note that this criterion became more restrictive over time since initially tumor properties were assigned to the entire CTV, leading to a high electrical conductivity in the entire target and hence also a high predicted SAR. Later, the planning procedure was improved by assigning only a specifically delineated GTV tumor properties and hence the SAR on average was reduced in the CTV in some cases leading to substantially lower TC_25%_ values.

### Pre-treatment planning controlled deep hyperthermia using VEDO

To be able to use the simulations also for dosimetry during treatment, we implemented the possibility to show the estimated SAR into VEDO [36]. This SAR is calculated using the measured signals (power and phase) applied to each of the twelve channels multiplied by the respective electric field per antenna as simulated in pre-treatment planning. Note that, this step assumes a perfect SAR distribution prediction, i.e. perfect arrangement modelling and no impact of non-modelled radiation losses in the waterbolus, which stresses the need for sound quality assurance.

By displaying the estimated SAR on top of the image data (Fig. 3), the technician has a much better insight in the applied complex interference patterns. This provides the means for correlating locations of high SAR to hotspots, i.e. high measured temperatures and/or complaints indicated by the patient.

#### Preplan adaptive hyperthermia

Initially, a library of three to six SAR distributions were computed prior to treatment, which were updated between treatments, to enable responding to high measured temperatures and patient complaints. This procedure required to estimate the possible complaints prior to treatment. Early experience showed that the SAR predictions not always correlated with pain complaints indicated by the patient. Therefore, we replaced this procedure by our dedicated complaint-adaptive treatment protocol for pre-plan adaptive application of HT [53]. In this protocol, complaints by the patient or high temperatures in normal tissue are converted by the operator into regions where the SAR level should be decreased. This strategy was implemented into VEDO to reduce the complexity of SAR-steering. By visualizing the SAR on top of the CT of the planning, locations of complaints can be elegantly discriminated. In addition, VEDO incorporates the information of the SAR applied to the target region. Hence, this approach provides a quantitative basis for steering action and reduces their dependence on the operator.

**Fig. 4:**
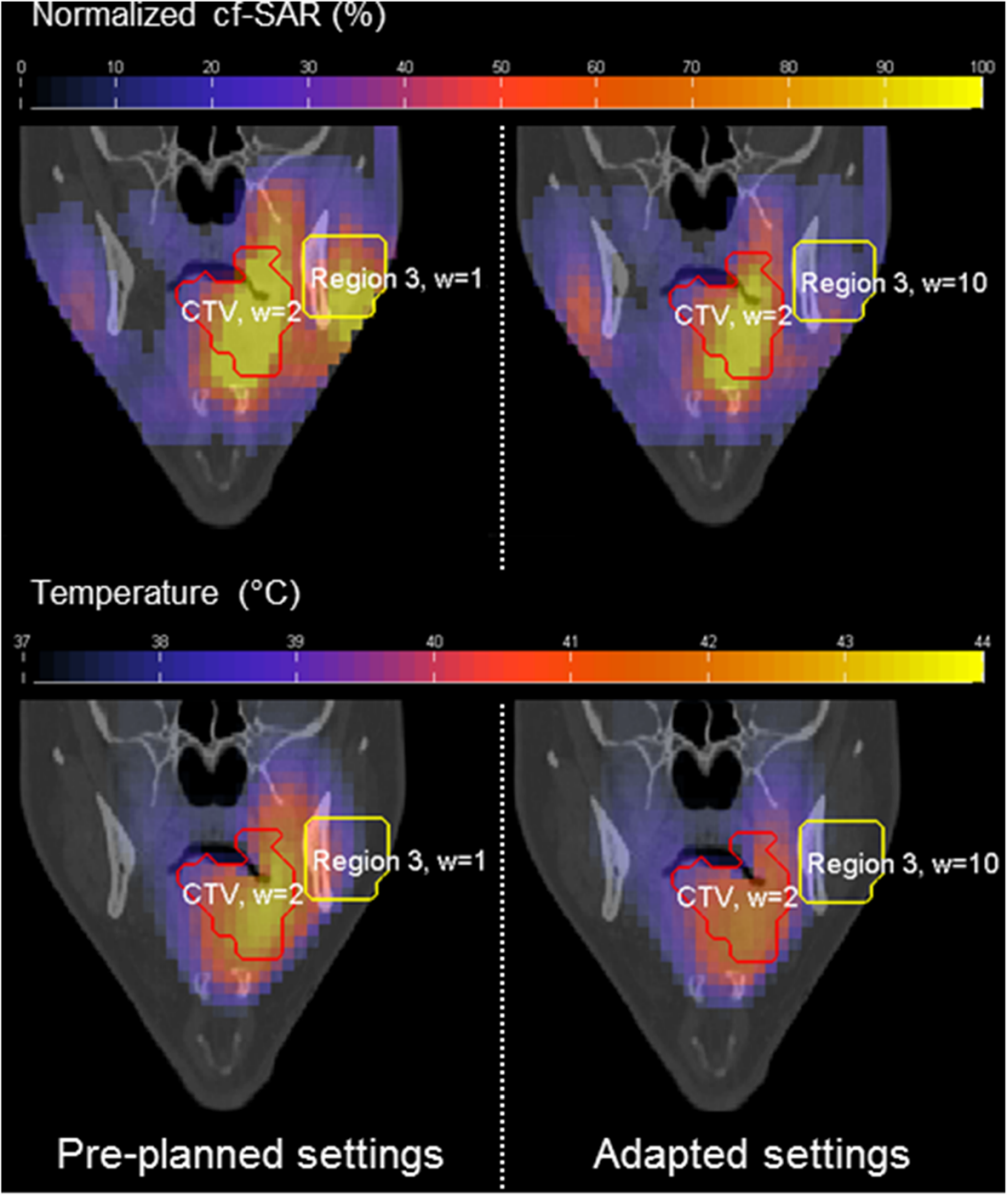
Coronal views in VEDO of the predicted normalized cubic-filtered SAR (%) and temperature (°C) distributions displayed as overlay over the CT scan. This figure shows the effectiveness of real-time adaptive treatment by adjusting the pre-optimized power absorption distributions. Re-optimization reduced the SAR at the hotspot (Region 3, yellow contour) by 2.3-fold and the maximum temperature by 2.2 °C, while the predicted temperature in the entire clinical target volume (CTV, red contour) was virtually unaffected, i.e. T90 reduced from 39.6 °C to 39.5 °C. Note that, following this effective re-optimization, increase in power can be used to increase the temperatures in the target region

Figure 4 shows the effectivity of the complaint adaptive procedure in VEDO when used during H&N HT. The coverage of the SAR pattern over the clinical target volume (CTV, red contour) optimized using HTP, led to a complaint at the left side of the jaw. After re-optimization, increasing the weight of Region 3 (yellow contour), the SAR level at the tumor reduced slightly (15 %) but the power could be further increased, resulting in enhanced power deposition in the tumor. This example also indicates that the on-line optimization approach is also an effective tool to respond on hot-spots related to the patient specific blood perfusion pattern, as at locations with low perfusion a moderate SAR will result in high temperatures. With on-line SAR optimization, the heat at these hot-spots can be reduced immediately with still maximum energy directed to the tumor.

### Current treatment procedure

#### Preparations and treatment planning

Initially inclusion for HT treatment is determined by the multi-disciplinary H&N tumor board. Next pre-treatment HTP is performed for decision making, used the thresholds defined before. Next the simulation results are presented and discussed in the RT board.

#### Treatment and QA

Following the clinical introduction of the HYPERcollar3D in 2014, this applicator is being used. Pre-treatment planning guided HT is applied with this device up to patients tolerance for 60 min after a heating up period of 15 min, aimed at achieving 40–43 °C throughout the target region. Patients tolerance is defined as temperatures in normal tissue above 44 °C or pain-related discomfort indicated by the patient. In current clinical practice, HT is added once a week to RT applied to the primary tumor and neck levels. To maximize the heat-induced sensitization in tumor cells while avoiding sensitization in normal cells [14], HT treatments are preferably applied one hour after irradiation, but the time-spacing is always within three hours.

Whenever possible, closed-tip catheters are placed interstitially under local anesthesia by a H&N surgeon, in the tumor and/or hotspot area [35]. Additional thermometry probes are placed on the skin. After catheter placement, a CT scan (“thermometry CT”) is made to document the location of all catheter tracts. In the HT treatment room, the patient is placed on an ordinary patient bed. A recently-implemented laser guided procedure is used to match patient positioning to the planning CT. Just before treatment, optical thermometry fibers are inserted in these catheters, for monitoring temperature in the tumor or for monitoring temperature in surrounding normal tissues, e.g. organs at risk, hotspots, skin. After thermometry probes are inserted, their exact insertion length is documented. Combined with the documented catheter tracks of the thermometry CT, this allows labeling temperature measurement locations, e.g. “tumor”, “muscle”, “fat” and rigorous comparison of measurement and simulated temperatures at exactly the same location in the patient. Additional thermometers are placed at the inflow and outflow of the waterbolus. After temperature sensor placement, the applicator is placed around the target volume. Then, the water bolus is filled with demineralized water, which is circulated at a temperature in the range of 20–30 °C, depending on target depth. During treatment, heart rate, power and phase per channel and the measured temperatures are continuously monitored and stored. After treatment, the fiber probes and the interstitial catheters are removed.

### Early clinical results

#### Heating quality with the HYPERcollar

The HYPERcollar applicator has been used in 48 HT treatment series administered to 47 patients with tumors in the oral cavity, nasopharynx, oropharynx, larynx, thyroid gland, trachea, hypopharynx and paranasal sinuses. For all patients, extensive 3 dimensional (3D) treatment plans were made using SEMCAD-X to find the optimal position of the applicator as well as the optimal settings of phase and amplitude per separate channel.

If their exact position is known at the mm-level, thermometers placed in catheters inserted in tissue feature the most accurate source of dosimetry data in H&N HT treatments. However, the catheters and their placement often are a burden to the patient and sometimes cause morbidity. Furthermore, invasive thermometry in many patients was not applied since the risks outweighed the benefits [54]. Therefore, from the initiation of the project, we focused at translating the setup from HTP towards the clinic and reproduce it in every session in order to enable reliable optimization of the SAR patterns using HTP. In Paulides et al. [35] we showed that maximizing the SAR in the tumor is effective when striving for higher temperatures, i.e. for the three analyzed patients high SAR values indeed correspond to high temperatures (R^2^ = 0.59–0.94). Note that there are inherent differences between SAR and temperature. In addition, the uncertainties will be reduced by the novel applicator design. The remaining sources of uncertainty, especially the thermal properties and their variation, still need to be addressed thoroughly for obtaining predictive simulations also at the temperature level.

#### Clinical outcome using the HYPERcollar

In 2014, we analyzed the treatments of the subgroup of patients with tumors that are considered to be in the traditional H&N regions (27 patients and in total 119 treatments of one hour). The target was the CTV of RT and the median CTV size was 63.5 ml. Delivery of power occasionally beyond 1 kW and estimated SAR levels (according to [36]) of on average 72.6 W/kg did not lead to achieving 43 °C. In the 16 patients where interstitial thermometry could be applied, we measured target temperatures up to 38.1–42.3 °C. Hence SAR levels up to four-fold of the SAR delivered in the pelvic region (~16 W/kg) still in cases produced unsatisfactory temperatures, which is a strong indication of the tremendous thermoregulatory response in the H&N region as compared to other regions. Excluding the three patients that were treated in a post-operative setting, a response rate of 53 % was obtained [54]. This response is very promising considering the learning curve and the fact that this was a very unfavorable patient group, i.e. 33 % locally advanced and 67 % re-irradiation. Importantly, no severe complications or enhanced thermal or mucosal toxicities were observed.

#### Preliminary analysis of heating quality with the HYPERcollar3D

Aimed at improving the median temperatures overall, but specifically for the nasopharynx and paranasal sinuses, we recently introduced the HYPERcollar3D into the clinic. The first six patients treated with the novel HYPERcollar3D were applied to patients suffering from tumors of the nasopharynx [1], base of the tongue [3] and melanoma in the parotic region [2, 55]. In these patients, a mean forward power of 280 W (range 161–366 W) could be applied. This exceeded our expectations since the reduced waterbolus of the HYPERcollar3D makes the applicator two times more efficient in power delivery and the general average for the HYPERcollar had been 400 W [39]. Expressed in the estimated SAR, which is calculated using the realtime measured powers/phases of the signals and pre-calculated electric fields [36], on average around 72–328 W/kg was achieved. Since the measured long-term average SAR with the HYPERcollar was 75 W/kg, these data support the theoretically predicted doubling of the applied SAR after replacing the HYPERcollar by the HYPERcollar3D [39]. Invasive thermometry could be applied in patient three and four. These data indicate that SAR levels converted into mean temperatures of 41.2 °C in the tumor (third patient) and 40.0 °C near the tumor (fourth patient), but also up to 42.8 °C in the masseter region near the tumor [55].

## Discussion

Early analysis indicates that, despite the fact that a learning curve was observed, the HYPERcollar3D provides a much better heating compared to the HYPERcollar. Note that due to the learning curve, more data are required for definite conclusions about the performance of the HYPERcollar3D using temperatures measured by invasively placed thermo-probes. In addition, the HYPERcollar3D provides a much more reproducible treatment setup, i.e. improved positioning, more reproducible waterbolus shape. We expect that this will improve the predictive value of the simulations, and will make our simulation adaptive HT approach more effective. Based on the predicted and measured increases in SAR and temperatures, we anticipate that the predicted increase in heating quality indeed converted into improved heating quality. Following the dose–response relations demonstrated for other sites [56, 57], we expect that this will also convert into improved clinical results.

In our work on deep H&N HT, we found that HTP plays a pivotal role. First of all, it provides the tool required to quantitatively optimize treatment settings, which inevitable to achieve sufficient treatment quality for HT applicators with over six independent channels. This requirement is even more pronounced when these devices are applied in regions with an irregular outer tissue contour where approximating the body as a homogeneous cylinder does not hold. In addition, HTP provides a quantitative basis for multi-disciplinary discussions to decide on inclusion, risk assessments, etc. Before treatment, we use the predicted SAR distributions to analyze the projected treatment quality and risk for side effects. The positioning alignment in the HYPERcollar3D further allows to match the RT and HT plans to assess if enhanced RT toxicity is likely in this patient. Simulation studies also showed that patients with a tracheostoma can be treated with the HYPERcollar, as long as the tumor is not located caudally of the tracheostoma. This was confirmed by treatment of 2 patients with a tracheostoma, without positioning problems and side effects, and TC_25%_ was sufficiently high (57 % and 64 % in these two patients). In addition, treatment planning provided the tool to investigate, and circumvent, the negative effects of metal in or near the target on the temperature distribution. Metal is standardly delineated in the treatment planning process, and hence the effect on the electric field distribution is taken into account. Clinical experience confirmed that toxicity can be prevented when this procedure is properly followed. In summary, HTP plays a pivotal role in our current treatment procedure. HTP allows to up front determine treatment feasibility and risks, it stimulates cross disciplinary discussions and it provides the means to optimize treatment settings before and during HT application.

In the current clinical practice, fiber-optic probes inserted into closed-tip catheters are used for assessment of the thermal dose. Initially, in our phase I study, intraluminal thermometry, i.e. catheters placed in the oral cavity or esophagus, was envisaged for monitoring target indicative temperatures. This procedure was used in the majority of phase III trials conducted in the H&N. For deep pelvic HT, for example, intraluminal measurements correlated with interstitial measurements. However, already in the first treatment, we found that such surface measurements in the H&N region are severely affected by breathing, swallowing and variable tissue contact. We therefore concluded that intraluminal measurements provide poor dosimetry and consequently we abandoned this method. Since then, we have used only invasively placed catheters in which the optical thermometers are inserted. These are not affected by poor tissue contact and hence provide the most accurate source of information. Unfortunately, invasive thermometry provides very limited spatial information due to their limited number. In addition, the sensor-catheters can usually remain only one or two sessions [42]. Therefore we developed HTP based dosimetry, which provides good accuracy in SAR predictions but fail in predicting the temperature [45]. The strongly dynamic response of tissues hampers the predictability of the temperature distribution. Hypothesizing that this dynamic response is strong but relatively stable across heated tissues, we studied if invasive temperature measurements could be exploited to adapt the 3D temperature simulations to match the measurements. In a novel iterative tissue property reconstruction procedure [54], we showed that optimized perfusion and conductivity values lead to a substantial improvement in simulation accuracy compared with the accuracy for literature values, i.e. the average absolute difference reduced from 12.0 ± 11.7 °C (baseline values) to 0.0 ± 2.1 °C [54]. In addition, we showed that the T50 can be predicted with a median accuracy of 0.4 °C for subsequent HT sessions when applying thermal tissue property reconstruction to invasive thermometry data of the first HT session [58]. Hence, our pre-plan adaptive 3D treatment planning provides the means to extrapolate sparse thermometry into good estimates of the complete 3D temperature distribution. This step is mandatory for guiding highly focused target conformal delivering of a safe and effective treatment.

## Conclusions and outlook

There is now a strong evidence base for applying HT in the H&N region. The five randomized studies all showed significant increases in complete response and/or overall survival, although in one study only in subgroup analysis [28]. Combining four of the randomized and four controlled non-randomized studies leads to a significant benefit in favor of the addition of HT, with an odds ratio of even 3.71. Despite these very encouraging results, unfortunately the monitoring of treatment quality has been suboptimal in these studies. Moreover, the time between application of RT, chemotherapy and HT was not always recorded. From pre-clinical work, we know that the temperature and time-delay both provide crucial factors determining the heat-induced sensitization of tumor cells for RT, and hence clinical outcome [13, 29]. Clinical establishing this pre-clinically determined relation critically relies on the scoring of these factors. Hence, clinical trials with accurate quality control during application of hyperthermia are required to investigate the clinical impact of sequencing and temperature. These data may provide options to further optimize the already impressive clinical results. As such, treatment control is pivotal for further developing H&N HT. The development of the HYPERcollar3D in combination with treatment planning control has shown to provide the required tools for accurate control and may enable studies to elucidate the parameters crucial for further improving clinical results.

Improvements in control beyond the state-of-the-art is envisaged by the development of magnetic imaging (MR) guided focused microwave HT, either hybrid [59] or truly integrated [60]. In early work, we already showed the feasibility of this step [59] as well as the accuracy of MR thermometry in neck equivalent gel phantoms [61]. In addition, a recent porcine study showed the feasibility of MR thermometry in superficial locations in the neck in vivo in the presence of motion artifacts [62]. Hence, this approach may provide the technology to non-invasively provide the dose control required to maximize treatment outcome. In addition, the treatment planning provides the means to perform pre-treatment planning, to adapt treatment settings in feedback control, and to extrapolate the measurements to locations outside the field-of-view.
